# Oleogels: Uses, Applications, and Potential in the Food Industry

**DOI:** 10.3390/gels11070563

**Published:** 2025-07-21

**Authors:** Abraham A. Abe, Iolinda Aiello, Cesare Oliviero Rossi, Paolino Caputo

**Affiliations:** 1 INSTM—Consorzio Interuniversitario Nazionale per la Scienza e Tecnologia dei Materiali, Via G. Giusti, 9, 50121 Firenze, Italy; abraham.abe@unical.it (A.A.A.); iolinda.aiello@unical.it (I.A.); paolino.caputo@unical.it (P.C.); 2 MAT-InLAB, LASCAMM CR-INSTM, Dipartimento di Chimica e Tecnologie Chimiche, Università Della Calabria, Ponte Pietro Bucci Cubo 14C, 87036 Arcavacata di Rende, Italy; 3 Department of Chemistry and Chemical Technologies, University of Calabria, Via P. Bucci, Cubo 14/D, 87036 Rende, Italy

**Keywords:** gel, organogel, food, mechanisms, applications

## Abstract

Oleogels are a subclass of organogels that present a healthier alternative to traditional saturated and trans solid fats in food products. The unique structure and composition that oleogels possess make them able to provide desirable sensory and textural features to a range of food products, such as baked goods, processed meats, dairy products, and confectionery, while also improving the nutritional profiles of these food products. The fact that oleogels have the potential to bring about healthier food products, thereby contributing to a better diet, makes interest in the subject ever-increasing, especially due to the global issue of obesity and related health issues. Research studies have demonstrated that oleogels can effectively replace conventional fats without compromising flavor or texture. The use of plant-based gelators brings about a reduction in saturated fat content, as well as aligns with consumer demands for clean-label and sustainable food options. Oleogels minimize oil migration in foods due to their high oil-binding capacity, which in turn enhances food product shelf life and stability. Although oleogels are highly advantageous, their adoption in the food industry presents challenges, such as oil stability, sensory acceptance, and the scalability of production processes. Concerns such as mixed consumer perceptions of taste and mouthfeel and oxidative stability during processing and storage evidence the need for further research to optimize oleogel formulations. Addressing these limitations is fundamental for amplifying the use of oleogels and fulfilling their promise as a sustainable and healthier fat alternative in food products. As the oleogel industry continues to evolve, future research directions will focus on enhancing understanding of their properties, improving sensory evaluations, addressing regulatory challenges, and promoting sustainable production practices. The present report summarizes and updates the state-of-the-art about the structure, the properties, and the applications of oleogels in the food industry to highlight their full potential.

## 1. Introduction

**Figure 1:**
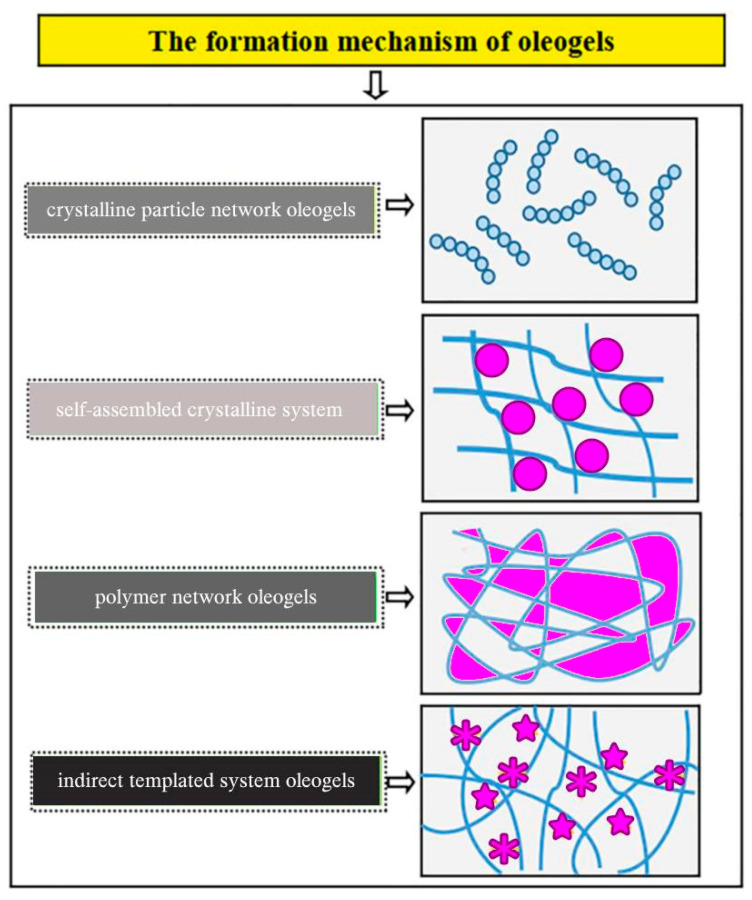
The mechanism of formation of oleogels [9].

Oleogels are structured edible fats formed by the gelation of oils. These structured lipid systems are formed via the self-assembly of low molecular weight compounds into crystalline fibers, which eventually lead to the gelation of an organic liquid, i.e., the oils. These compounds, known as gelators, are surfactant-like molecules or polymers in oil, such as fatty alcohols, waxes, and lecithin, that create a three-dimensional network which, in turn, immobilizes the oil [1,2]. The self-assembly process (see Figure 1) occurs at low gelator concentrations (of about 2%), thus making them a very efficient alternative to conventional fats [3]. The reorganization of these gelators upon heating and cooling physically entraps the oil, which leads to the formation of a semi-solid structure in the presence of low amounts of saturated fatty acids. One fascinating property of these semi-solid oleogels is their ability to retain the nutritional profile of the base oil, making them highly efficient and versatile in food applications, such as fat replacement [4,5]. It is worth noting that they can also have non-food applications, such as the encapsulation and controlled release of bioactive compounds and the formulation of cosmetic products to prevent oil leakage. They are also used in the plastic and paint industries, where they are useful to entrap organic solvents [6,7,8].

Although oleogels have a variety of applications in different sectors, they are most widely used in the food industry as oil structuring agents and fat replacers to develop healthier food products due to the legislative limitations regarding the use of trans fatty acids [10,11]. Oleogels have a huge potential in the food industry as they can be used to improve the nutritional profile of food and can also be used as a fat mimetic in the replacement of solid fats in food products via oil structuring. They can also be used in meat analogues and plant-based dairy products to enhance the texture and mouthfeel, thus providing a healthier alternative without compromising the sensory experience [12]. The ever-increasing demand for the use of oleogels in the food industry is due to the health-driven demand for low-trans, low saturated fat products, the ability of the oleogel-based food to retain desired texture and sensory properties without harmful fats, and the compatibility of these oleogel-based food products with clean label and vegetarian/vegan trends [13].

Fats and oils are basically triglycerides made up of monounsaturated, polyunsaturated, and saturated fatty acids. Among these fatty acid types, only trans and saturated fatty acids have been implicated in chronic disorders, such as cardiovascular diseases and type 2 diabetes [3,10]. The bone of contention, however, lies in the fact that these very fatty acids, known to be harmful to human health, are also the ones that give food its distinctive flavor and texture. This is due to the triglycerides in these fatty acids assembling to form a supracolloidal network, transforming fats into solid or semi-solid materials, which give fatty foods their characteristic structure and mouthfeel [14]. In fact, the World Health Organization (WHO) put forward a recommended fat limit regarding the total energy intake for each of these fatty acid classes: 30%, 10%, and 1% for total, saturated, and trans fat, respectively [15]. In the European Union (EU), the European Commission imposed a restriction on the use of trans fats to 2 g per 100 g of total fat, as stated in Regulation No. 649 from 24 April 2019 [16]. These regulations, amongst others, which were set up in place for food safety, brought about the need for food engineers and scientists to develop solutions that would constitute the replacement of trans and saturated fatty acids with healthier alternatives, without altering the physicochemical properties of the food products. All this, while also maintaining important qualities, such as flavor and texture, that consumers appreciate. Oleogelation was found to be a suitable and effective technique for achieving this feat, and in recent years, advancements in this technology have seen the food industry make strides towards food product safety and optimization. Overall, oleogels are proving to be pivotal not only for the advancement of the food industry but also in other sectors, such as pharmaceuticals, cosmetics, and lubricants. Ongoing research continues to explore additional uses of oleogels in food-grade lubricants and bio-based packaging materials, highlighting their potential for sustainable and health-oriented food production practices [17,18].

This review is aimed at highlighting the various uses, key applications, functional/physicochemical properties, and benefits of oleogels. We also take a look at the limitations faced by these semi-solid materials in food applications, and the possible trends that oleogel applications in food products might follow in the near future.

## 2. Fundamentals and Applicability of Oleogels in Food

### 2.1. Classification

#### 2.1.1. Oleogels vs. Hydrogels

**Figure 2:**
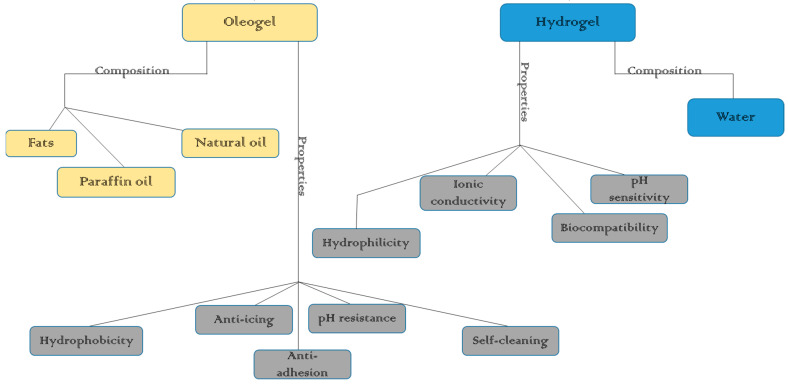
Oleogels vs. hydrogels.

In the food industry, other organogels can be used as structuring agents. Hydrogels have been around for longer and can serve the same purpose. Actually, hydrogels have been researched more than oleogels, and more is known about their structure, functionality, and application. Figure 2 illustrates a few characteristics of oleogels and hydrogels. Oleogels and hydrogels are two distinct types of gels that can be used to modify the texture, nutritional profile, and stability of various food products. Although similar, they differ significantly in composition, formation mechanisms, properties, and applications (Figure 2). They both respond to various physical and chemical stimuli such as pH, temperature, light, and mechanical deformation [19].

Oleogels have organic liquids (oils) in the liquid phase and can absorb and release substances and swell and retain large quantities of the liquid phase (up to about 99%). The combination of properties conferred by the organic liquid and gelator components gives oleogels a wide range of varied functionalities across industries, especially in the food sector. Hydrogels, on the other hand, are more popular, have been more deeply researched, and are more biocompatible, with natural tissue-mimicking properties due to the fact that they contain large amounts of water and aqueous solutions. The fact that hydrogels are water-based limits their choice as gelators and also brings about their low affinity to hydrophobic compounds. In addition, hydrogels have low environmental and thermal stability [20,21,22,23,24].

Oleogels have better applicability than hydrogels in food products because there is a wider range in the choice of the liquid phase for oleogels, and this can be exploited to provide beneficial properties to the products in which oleogels are used. Oils are more thermally stable than water, and the thermal stability of an oleogel can be manipulated and improved using the boiling point of the solvent (oil)—a feat which is difficult to achieve in the case of hydrogels, since they are water-based [25,26]. Other features such as ice-phobicity, hydrophobicity, and anti-biofouling properties can also be improved by swelling the oleogels with lubricants, thus creating environmentally stable gels [27,28,29,30,31,32].

#### 2.1.2. Oleogelator Type

Oleogels can be classified on the basis of gelators. In Section 2.5, oleogels will be divided into classes based on the nature of their gelator/gelling agent. In this section, however, gelators will be distinguished according to their molecular weight.

**Figure 3:**
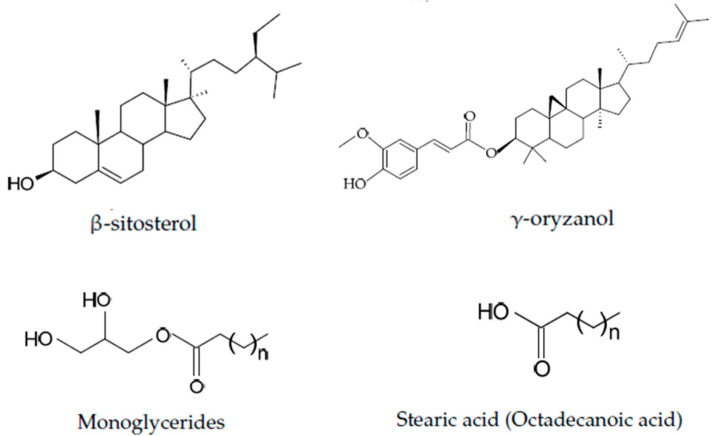
Chemical structures of a few low molecular weight gelators.

**Figure 4:**
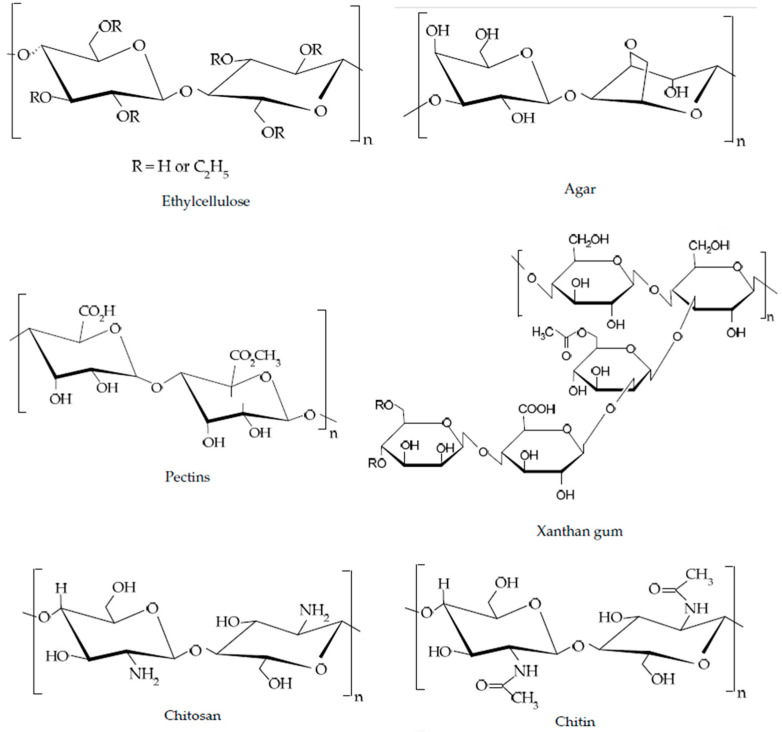
Chemical structures of common high molecular weight gelators.

▪ *Low Molecular Weight Gelators (LMWGs):*
  These are mostly lipid-based small molecules, such as mono and diglycerides, that self-assemble into networks capable of immobilizing oils [33]. Fatty acids and fatty alcohols, such as glyceryl monostearate, are one of the main types of LMWGs [34]. Another group of LMWGs is phytosterols and their derivatives, such as phytosterol esters [35]. Compounds such as β-sitosterol and γ-oryzanol are typical examples of sterols and their derivatives. These compounds assemble to form fibrillar networks, providing structural stability to the resulting oleogels [36]. Other LMWGs are natural waxes, such as rice bran wax and beeswax, which crystallize and form networks within the oils [37]. The thermal properties and microstructure of these oleogels play a critical role in determining their firmness and spreadability. The chemical structures of a few LMWGs are shown in Figure 3.
  - Waxes:
    Waxes are low molecular weight oleogelators that are obtained from plants or animal sources via biosynthesis and are in the form of crystalline solids. Substantially, waxes are mixtures of medium- or long-chain fatty acids and long-chain fatty alcohols in free or esterified form (wax esters). Examples of waxes commonly used as LMWGs are beeswax, carnauba wax, rice bran wax, and sunflower wax [38,39,40,41].
  - Phytosterols and phytosterol esters:
    This group of LMWGs is a large group of steroid-type alcohols that occur in free or esterified forms and are only biosynthesized in plants [42]. Examples of phytosterols and their esters are β-sitosterol [43] and γ-oryzanol [44].
  - Fatty acids and monoglycerides:
    These are groups of oleogelators that form different molecular arrangements due to their different chemical groups and structures, which bring about the various mechanical and textural properties in oleogels. Free fatty acids, such as oleic acid, stearic acid, and palmitic acid, and monoglycerides, such as monopalmitin, monostearin, and glycerol monolaurate, can be used to create stable gels in lipid-based systems [45]
▪ *High Molecular Weight Gelators:*
  This group of gelators self-entangle or cross-link to form networks capable of immobilizing oils and are either polymer-based or polysaccharide-based. A common example is ethylcellulose—a food-grade polymer that dissolves in oil at high temperatures and forms a gel when cooled. Ethylcellulose-based oleogels have been utilized to replace solid fats in various food applications, offering desirable mechanical properties and thermal stability [8]. Polymer-based oleogels are formulated using synthetic or natural polymers as gelling agents. They usually exhibit different rheological properties compared to lipid-based oleogels, hence bringing about a more versatile range of textures and structural integrity. The viscoelasticity of this class of oleogels can be tuned through the concentration and type of polymer used, allowing for customizable applications in the food sector [46]. Other examples of high molecular weight gelling agents (HMWGs) are based on polysaccharides and proteins, which entrap oils and bring about oleogels with specific viscoelastic properties. These gelators form three-dimensional networks, mostly via hydrogen bonding [34]. The chemical structures of a few HMWGs are shown in Figure 4.
  - Polysaccharides:
    Polysaccharides are widely used in various industries due to their cost-effectiveness, non-toxicity, and wide consumer acceptance. Hydrophilic polysaccharides are Generally Recognized as Safe (GRAS) food additives and are used in the formulation of oleogels. This group of HMWGs can be obtained from a number of sources, such as plants, animals, algae, and microbes. Examples of polysaccharides used in oleogel formulation are agar [47], pectin [48], xanthan gum [49], ethylcellulose [50], Chitin, and Chitosan [51,52].
  - Proteins:
    This group of oleogelators is classified based on shape: globular proteins and fibrous proteins. The former form a gel-like structure via their ability to create cross-links or aggregates [53], while the latter form gels when they are heated and then cooled. Examples of protein oleogelators are whey protein, zein, soy protein, and gelatin [54].
▪ *Mixed component (hybrid) gelators:*
  Combining different types of gelators can bring about a hybrid effect, exploiting the best properties of each component gelator, thus enhancing the properties of the oleogels [55,56]. For example, mixtures of monoglycerides and methylcellulose have been shown to influence crystallization behavior and rheological properties, allowing for tailored textural attributes in oleogels and ultimately resulting in oleogels that could be used as base stock for the production of shortening and margarine [57].

### 2.2. Mechanism of Oleogel Formation

**Figure 5:**
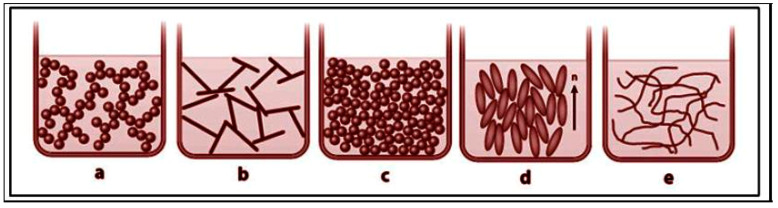
Schematic illustration of different strategies of oleogel formation [3]. (**a**) Crystalline particle, (**b**) fibril network, (**c**) particle fillers, (**d**) liquid crystalline mesophase, and (**e**) polymer network.

Oleogels are formed by the self-assembly of gelators, and this self-assembly can occur in one of two ways—either through the formation of crystallites or through the formation of self-assembled fibrillar networks (SAFINs) (Figure 5). In the former, certain gelators, such as fatty acids, fatty alcohols, and waxes, crystallize when cooled, bringing about a molten state, thus forming a network of crystalline networks and structures that trap the liquid oil. This process involves nucleation and crystal growth, leading to a solid-like network within the oil matrix [12]. Other gelators, such as lecithin, can form oleogels through the self-assembly of their amphiphilic molecules [58,59]. Due to the peculiarity possessed by amphiphilic molecules having simultaneously polar and apolar moieties within their molecular structure, they are able to form extended networks and complex structures. This principle is true in general. Even at longer length scales, the complexity of amphiphilicity is a rule: amphiphilic block copolymers, for instance, follow, in fact, the same mechanism, with self-aggregation forming the template for porous structures [60].

Research studies have shown that 3D mechanistic modeling techniques that examine the microscale structure of these crystalline lipid-based materials obtained through non-destructive imaging can be used to mathematically model their mechanical properties [61].

On the other hand, SAFINs are formed when LMWGs, such as sterols and certain glycolipids, self-assemble into fibrillar structures via non-covalent interactions. These fibrils entangle to form a three-dimensional network that immobilizes the oil [62]. The networks formed during oleogel formation are often driven by non-covalent intermolecular interactions, such as hydrogen bonding, van der Waals forces, and π-π stacking, which can occur between the gelator molecules or between the gelator and the oil components [2]. In this regard, it is worth noting that the resulting structure is dictated by a delicate equilibrium between all these kinds of interactions, including eventual H-bonds, as well as steric hindrance, as model surfactant-based liquid mixtures have recently clarified [63].

### 2.3. Physicochemical Factors Influencing Gelation

1. **Solubility and Self-Assembly**: For gelation to occur, the molecules of the gelator need to dissolve well within the oil and then self-assemble to form a three-dimensional network that traps the oil molecules. If the gelator has a lower solubility in vegetable oil, there will not be enough gelator molecules dispersed to form a sufficient network [64]. Aromatic Imino Acids (AIAs), which are small organic molecules containing aromatic rings and imino groups (–C=NH or –NH2), are one of the groups of molecules that facilitate self-assembly into ordered supramolecular structures. The way these AIA molecules interact with each other (their self-assembly behavior) might be hampered in vegetable oil, hindering network formation [65].
2. **Chemical Interactions**: The gelator molecules likely interact with the oil molecules through various forces like hydrogen bonding, van der Waals forces, and π-π interactions (interactions between aromatic groups). The presence of functional groups in vegetable oil that can compete with these interactions can disrupt the formation of a strong gelator network. For instance, if the vegetable oil has a high content of fatty acids with free carboxylic acid groups, they might compete with the hydrogen bonding between gelator molecules, weakening the gel network [66]
3. **Oil Compositional Complexity**: Vegetable oils are triglycerides composed of various fatty acids. This diversity in fatty acid chain lengths and the presence of different functional groups within the vegetable oil can lead to steric hindrance and hinder the formation of a uniform gelator network throughout the oil [64].
4. **Shear Sensitivity**: The gelation process itself or the properties of the oil might affect the gelator network’s susceptibility to shear, leading to a weak or disrupted gel [65]. The stir rate can also improve the solubility of the organogel in the oil. In a case study, when the rate was increased, it affected crystallization onset even though a lower viscosity was observed [67].

### 2.4. Advantages of Oleogels in Food Applications

The ability of oleogels to structure oils into semi-solid forms makes them suitable for various food applications, as will be discussed in this section. One main application is the enhancement of texture and stability during the storage of food products. An example of stability enhancement for food product storage is oleogels used in marinades to prevent oil separation [2,12]. In addition, lipid-based oleogels can have mechanical qualities similar to those of conventional shortening, with increased hardness and chewiness due to aggregate–aggregate interactions that form a three-dimensional network, according to a study by Almeida and Bahia [66]. This structuring brings about an improvement in the overall food texture due to the prevention of excessive crosslinking that usually occurs in pure oil systems.

▪ **Oleogels as healthier fat alternatives:**
  Due to the risks saturated and trans fats pose to human health, a very significant goal of food scientists and formulators over the years has been to replace these fats with healthier unsaturated fats. The use of oleogels has made this possible, as they enable oils in unsaturated fatty acids to structure themselves into solid-like consistencies, hence facilitating their incorporation into food products that conventionally rely on solid fats. This application of oleogels brings about health benefits via the improvement of the lipid profile in the food product. For example, phytosterol-based oleogels have been shown to be capable of effectively replacing saturated fat in several contexts via the “Edible Oleogels for Reduction of Saturated Fat” project [68]. In 2021, a research study was conducted in which pork patty fat was replaced with an oleogel derived from linseed oil, rich in polyunsaturated fats (PUFA). The results obtained in the study demonstrated that replacing 25% and 75% of the solid fat with an 8% γ-oryzanol–β-sitosterol oleogel improved the fatty acid profile [12]. Although there is still room for improvement, oleogels have demonstrated enhanced nutritional value and oxidative stability in lipid-based foods, while preserving the desired sensory attributes [69].
▪ **Oleogels for the controlled release of bioactive compounds:**
  Oleogels serve as effective delivery systems for bioactive compounds, protecting sensitive ingredients from degradation and enabling controlled release. Their semi-solid matrix can encapsulate lipophilic bioactives, enhancing their stability and bioavailability. Recent studies have demonstrated the potential of oleogel-based systems in delivering functional molecules in food applications, highlighting their versatility in encapsulating various bioactive compounds [12,70,71,72].

### 2.5. Types of Oleogels Used in the Food Industry

As highlighted in Section 2.1, oleogels can be categorized into several system classes based on the type of gelling agents used:

▪ **Lipid-based Oleogel Systems:**
  These oleogels are obtained by forming a stable gel network with lipid-based, low molecular weight gelators. Lipid-based oleogels are semi-solid materials that are primarily composed of oils, which, via oleogelators—such as monoglycerides, waxes, and phospholipids—are structured into a gel-like network [69].
  Lipid-based oleogels are being investigated for use in pharmaceutical formulations for drug delivery systems, demonstrating their versatility beyond food applications. Opportunities for regulated release mechanisms and enhanced bioavailability of active compounds are presented by their capacity to encapsulate oils in a structured network [71,72]. Oleogels’ textural qualities make them appropriate for a range of food applications, such as spreads, baked goods, and dairy substitutes. For example, it has been demonstrated that adding oleogels to batter formulations increases air incorporation when compared to traditional oil batters, giving baked goods a lighter texture while maintaining the desired hardness and chewiness [37].
▪ **Protein-based Oleogel Systems**
  Because of their distinct structural and functional characteristics, protein-based oleogel systems are becoming important substitutes in the field of oleogels. Textural qualities can be greatly impacted by the gel-like structures that are created when proteins and oils interact. Although they frequently display a shorter linear viscoelastic region, research study results have shown that protein-templated oleogels can achieve viscoelastic behavior comparable to that of lipid-based oleogels [12]. This kind of oleogel is especially useful for creating healthier food options, like meat products, where protein-based oleogels can enhance health profiles without compromising texture by substituting saturated fats [73,74,75].
  Opportunities for innovation in product formulation and functionality are presented by the capacity to modify the characteristics of protein-based oleogels using various processing methods. One technique for formulating protein-based oleogels is the emulsion template method, in which artificial proteins act as emulsifiers to produce stable emulsions that solidify into oleogels when the aqueous phase is removed [76]. By exploiting proteins’ amphiphilic properties, which enable them to interact with both hydrophilic and hydrophobic substances, this technique improves the oleogel’s structural integrity [69]. Furthermore, a key characteristic for many food and pharmaceutical applications is the ability of protein-based oleogels to maintain their structure over temperature cycles due to their thermo-reversible behavior [77].
  The type of protein aggregates and how they interact with the oleogel matrix affect the mechanical characteristics of protein-based oleogels. Although a lot of research has been completed to better understand the relationship between structural and mechanical properties, most of the analysis has been performed indirectly, and occasionally destructively, using methods like optical microscopy and X-ray diffraction [78]. Non-destructive methods that can precisely depict the three-dimensional microstructure and offer a thorough mechanical characterization at the microscale level are still lacking. However, it is recognized that the rheological features of these oleogels can be greatly influenced by the presence of robust protein-based gel networks [77]. Protein-based oleogels are known for their enhanced nutritional profile and oxidative stability in addition to their structural benefits, which can improve the sensory aspects of lipid-based foods [10]. Because these oleogels help reduce fat without sacrificing the desired texture and mouthfeel, they can be used in a variety of products, including spreads, baked goods, and dairy substitutes [79].
▪ **Polymer-based Oleogel Systems:**
  Oleogels do this by using water-soluble food polymers [34]. These oleogels’ viscoelastic features are very different from those of lipid-based systems because they can be customized for particular uses and generally exhibit a higher level of structural stability [12]. To improve the overall integrity of the gel, polymer-based oleogels, for example, frequently have a longer linear viscoelastic region, indicating stronger interactions between the polymers and the oil [57]. Applications needing a stable structure, such as in processed foods, benefit greatly from these systems.
  The use of polymer-based oleogels in a variety of food products demonstrates their versatility. They can enhance texture without sacrificing flavor when used as fat substitutes in baked goods, sauces, and dressings. Compared to conventional oil-based batters, studies have shown that the addition of oleogels can improve air incorporation in batters, improving baked goods’ volume and texture [73]. Additionally, consumers looking for healthier options may benefit from the gel network created by these polymers as it may help control lipid release during digestion and promote a more stable physiological response, keeping the consumer full for longer, hence mitigating excessive food cravings and high calorie intake [79,80,81].
  Polysaccharides, which can be prehydrated and then mixed with hydrophobic oils in a two-step procedure, are commonly used in the composition of polymer-based oleogels. Oils can be physically trapped within the biopolymer matrix with this technique [81]. Agar, gellan, and xanthan gum are a few examples of the hydrocolloids (complex nondigestible polysaccharides that dissolve or disperse in water to give thickening or viscosity) that can be utilized as polymers. Each of these hydrocolloids contributes distinct rheological and thermal characteristics that affect the oleogel’s overall stability and functionality [73,74].
  The emulsion–templated approach is one of the most widely used techniques for creating polymer-based oleogels. First, a stable emulsion is created, followed by the removal of the aqueous phase. This indirect method makes it possible to create oleogels by producing a concentrated gel structure that retains the oil phase [74]. This method is particularly advantageous as it facilitates the creation of oleogels with controlled oil release properties, making them suitable for various applications, including confections and baked goods [79].
  In comparison to lipid-based and protein-based oleogels, polymer-based systems have a number of benefits, such as reduced costs and the possibility of simpler texture and mouthfeel modification. Further customization of the oleogel’s characteristics is made possible using water-soluble polymers, which enables producers to adapt their goods to particular dietary requirements or customer preferences [10,34]. As food producers constantly seek substitutes for saturated fats, polymer-based oleogels present a promising avenue for innovation in food formulation.
  The hybrid-based oleogel system is noteworthy when categorizing the different kinds of oleogels used in food. In contrast to their single-component counterparts, hybrid-based oleogels offer better textural qualities, stability, and nutritional profiles by combining two or more structuring agents, such as lipids, proteins, and polymers, to maximize the advantages of each system. Hybrid oleogels are a promising solution for particular problems in food formulation and product development because of their adaptable qualities. Lipids, which give the gel structure, are usually combined with proteins or polymers to create a matrix that enhances stability, texture, and release characteristics. By adding polymeric or protein-based gelators to lipid systems, the viscoelastic qualities of the oleogel are improved by forming a stronger network [13,34,82]. Bioactive substances, especially hydrophobic medications and nutraceuticals, are effectively transported by hybrid oleogels. The matrix components can be carefully chosen to maximize the controlled release of these compounds. Research has demonstrated that the physical characteristics of the lipid and polymer components in the gel can have a substantial impact on the release kinetics of bioactive substances, including β-carotene [12,79].

### 2.6. Applications of Oleogels in the Food Industry

**Figure 6:**
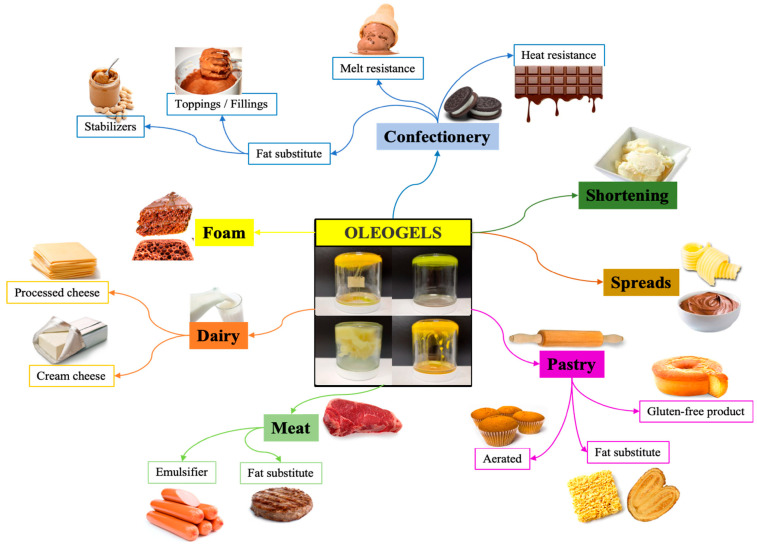
Application of oleogels in different food products [69].

Oleogels have a variety of applications in the food industry transcending different food products and food groups as shown in Figure 6 and Table 1. These applications are explained further in this section as follows.

▪ **Fat replacers in processed foods**
  Oleogels have become materials of interest as a result of their health benefits. As reported in the literature, oleogels considerably reduce lipid digestibility, thus lowering total fat consumption. As a result of this phenomenon, blood triglyceride and total cholesterol levels are reduced, which improves serum lipid profiles, consequently improving cardiovascular health [83]. According to one noteworthy study, participants who ate meals prepared with coconut oleogels showed lower blood triglyceride levels compared to people whose meals were prepared from regular coconut oil [84]. The structural characteristics of oleogels limit the absorption of dietary fats while delaying digestion, which reduces calorie intake. The mechanical resistance of oleogels reduces the rate of lipid lipolysis, evidencing their fat absorption reduction capacity in the digestive tract [9,85].
  Oleogels function as fat substitutes in processed foods by exploiting a range of structural and compositional characteristics that are very similar to those of conventional fats. As mentioned previously, oleogelators, such as ethylcellulose or hydroxypropyl methylcellulose, are used in combination with edible oils via techniques like melt blending or oil-in-water emulsions to form these gel-like structures [70,86]. So as to ensure that the oleogel maintains the desired consistency, the oil-trapping network, which constitutes the oleogel, is created using procedures such as vacuum drying or open-air evaporation to remove the solvents used during the oleogel preparation [86]. In products such as cream cheese, ice cream, and baked goods, the ability of oleogels to replicate the sensory features and mouthfeel of solid fats is fundamental to improve the overall consumer experience [69]. As a result of consumer preference for food with healthier profiles, manufacturers are now investigating oleogels as clean-label substitutes.
  The versatile nature of oleogels is recognized in a range of food products in which they improve stability and textural properties while retaining moisture. For example, in confectionery, oleogels improve melting properties and contribute to the prevention of fat bloom [69,87]. In bakery applications, they also provide moisture and softness to cookies and pastries [69,75,87].
  **Bakery products:**
  Oleogels have been successfully incorporated into a range of baked goods, including cakes, cookies, and pastries. Studies indicate that cookies formulated with oleogels can achieve comparable mouthfeel, taste, and overall acceptability to those prepared with conventional shortening [88,89]. Similarly, traditional fats in cake formulations can be replaced by oleogels without adverse effects on texture or flavor. Evidence suggests that oleogels act as effective binding agents in bakery products, enhancing stability and prolonging shelf life [9,90].
  **Confectionery:**
  The application of oleogels has extended to the confectionery sector, particularly in chocolate. Beta–sitosterol-based oleogels and other naturally derived systems have been investigated for their ability to produce healthier chocolate formulations while maintaining desirable sensory properties [10]. Additionally, oleogels have been utilized in chocolate fillings and spreads, where they improve product stability and serve as viable alternatives to hydrogenated fats [87,91]. Although at the moment, the legislation in most parts of the world does not allow for oleogels to be used in standard chocolate, oleogel-based chocolate products are allowed to be labeled as chocolate flavored or compound coating.
▪ **Edible coatings and encapsulation**
  Oleogels can also be used in the encapsulation of bioactive substances and creating edible coatings due to their specific qualities. As oleogels can be designed to mimic the sensory properties of conventional coatings, their texture is quite important in their applicability in food product development. Oleogels have been shown to improve emulsion stability—an essential property for the preservation of the integrity of food materials and the bioactive substances they contain, thus improving food quality during storage [69,92,93]. Research studies have demonstrated that not only do oleogels encapsulate higher loads of bioactive compounds, but they also facilitate their controlled, step-wise release during digestion [93,94].
  Even though oleogels provide many desirable properties in food materials, their relative production cost compared to conventional materials could prevent their widespread use [95,96]. In oleogel design, parameters such as chewiness, cohesiveness, and hardness are fundamental to achieve the desired texture of traditional coatings. The effectiveness of oleogels as edible coatings is greatly influenced by their textural characteristics [13,97]. One more fundamental factor that influences the performance of oleogels as edible coatings is emulsion stability. For the coating to stand the test of time regarding its effectiveness, fluid integrity must be maintained to ensure a consistent structure [69,92]. Research studies have reported that oleogels produced with certain oleogelators can improve emulsion stability, which enhances the bioactive substance encapsulation capacity of the oleogels, maintaining food quality in storage conditions [69,93]. Oleogels prolong the shelf life of products by serving as a barrier between oxygen and moisture, thus delivering the health benefits associated with the bioactive ingredients encapsulated therein [77,98].
  The three-dimensional structure of oleogels can effectively shield sensitive compounds, maintaining their effectiveness through the processing and storage periods of food production [13,94]. Oleogels have significant implications for health and nutrition, as their encapsulation capabilities help to improve the delivery and absorption of fat-soluble vitamins (A, D, E, and K), antioxidants, and probiotics, underlining their role in promoting health benefits. This is especially important as these vitamins are quite susceptible to oxidation and degradation when exposed to light, heat, and moisture [99,100]. Techniques such as microencapsulation and emulsion methods have been applied to optimize the performance of oleogels, and high encapsulation efficiencies and successful protection of bioactive compounds during gastrointestinal movement have been reported in the literature [101].
  Recent developments in encapsulation methods have shown that oleogels can shield vitamins, antioxidants, and omega-3 fatty acids from deterioration during processing and storage [46,102]. For example, a recent study effectively demonstrated how to encapsulate vitamin C in oleogels using a two-step emulsion–template method. The integrity and the structure of the vitamin were preserved due to the outstanding antioxidant qualities of the oleogels [103,104]. Recent research has focused on optimizing the formulation and processing techniques for oleogel-based systems to enhance their performance in encapsulation applications. Supercritical CO_2_-drying and freeze-drying are two new hydrocolloid combinations and processing methods that have been studied to improve the structural integrity and functional properties of oleogels [91,97]. Furthermore, studies have shown how important it is to select the appropriate oleogelators and processing parameters to create encapsulated products with the required properties [75,97].
  The application of oleogels as edible coatings and encapsulation agents presents a viable alternative for conventional petroleum-based materials, especially in the food sector. Encapsulation via oleogels has been shown to protect bioactive ingredients from sun exposure, heat degradation, and oxidation, bringing about their uniform dispersion in food products [105]. However, the commercialization of oleogel-based food products needs to be preceded by a few restrictions and knowledge gaps being addressed. Bridging these gaps is fundamental for the expansion and widespread consumer acceptance of oleogel technologies in the food sector [91,96,106,107].
▪ **Meat and dairy alternatives**
  By altering the texture of plant-based meat analogues and dairy substitutes, oleogels can be incorporated into their formulation. Oleogels can be made to mimic the textures of animal fat in processed meats and plant-based substitutes, increasing their mouthfeel and juiciness [108]. Much research has been performed over the years to produce oleogel shortenings from vegetable oils with high melting points, such as palm stearin and sunflower oil [109]. Because of the gelation process and the characteristics of the resulting oleogels, they are especially well-suited for structuring plant-based meat products, improving their distribution of fat and texture [110].
  Plant proteins possess a variety of properties essential for the successful implementation of oleogels. Due to their amphiphilic nature, they can interact effectively with both lipid and aqueous phases, thus contributing to emulsification, foaming, and gelation properties [111]. The structural properties of these proteins, such as their surface charge and the availability of reactive groups, influence the definition of the functionality of oleogels [112]. In order to optimize the use of oleogels in food applications, it is important to conduct more research to unravel the dynamics of the interaction between protein sequence, structure, and functionality. Some ongoing research via the utilization of emulsifiers in oleogel formulation has developed stable structures that improve the texture of plant-based meat substitutes [13]. To ensure the quality and functionality of oleogels in meat analogs, a thorough analysis of raw materials needs to be carried out. This usually involves measuring the moisture content, protein levels, fat content, and mineral composition. More in-depth techniques, such as near-infrared (NIR) spectroscopy and rheology measurements, provide valuable insights and key information on the processing behavior and stability of oleogels. These methodologies help to characterize the size distribution of fat replacers, which is vital for achieving the desired texture and sensory attributes in the final product [10,13].
  Developments in extrudable fat technology are reported in [112]. Manufacturers could produce more lifelike meat textures, like the fat marbling in steak, by mixing oleogels with other plant protein ingredients. This could greatly improve their entire eating experience, in addition to making plant-based meats more aesthetically pleasing [69,113]. Numerous real-world uses for oleogels have been studied in relation to plant-based meat substitutes. According to these studies, adding oleogels can enhance the sensory qualities of plant proteins, which frequently have unpleasant textures and off-flavors. Oleogels can improve overall palatability and cover up these off-flavors, giving customers a tastier experience [73,93]. Despite these benefits, obtaining the proper melting temperature gradient in oleogels remains a major challenge. This is necessary to replicate the sensory qualities of marbled meat, which calls for solid fat that melts at temperatures higher than room temperature [69,88]. Due to the growing demand for plant-based substitutes, the investigation of oleogels in plant-based meat formulations offers numerous chances for texture modification innovation and advancement. Furthermore, more investigation into how consumers view textural qualities will aid in improving oleogel formulations to suit particular tastes and increase marketability [114].

**Table 1:** Overview of the application of oleogels in different food groups.

Food Group	Oleogel Organic Phase	Gelator(s) Used	Result Obtained	Reference
Meat products	Canola oil	Ethylcellulose (8%, 10%, 12%, 14%) with or without sorbitan monostearate (1.5%, 3.5%)	Oleogel brought about a hardness value comparable to that of beef fat, analyzed via sensory and texture profile analysis.	[115]
	Linseed oil	γ-oryzanol and β-sitosterol	Successful 25% and 75% replacement of pork fat in meat patties. The textural parameters in terms of hardness, chewiness, and cohesiveness were the same with the control sample.	[116]
	Mixture of olive oil, fish oils, and linseed oils	11% beeswax	The oleogel use led to a 15% pork back fat reduction in pâtés. The sensory parameters of the oleogel and control sample were the same.	[117]
	Canola, soy, and flaxseed oils in addition to rendered beef fat	Ethylcellulose + 5% sorbitan monostearate	The hardness of meat batters was higher when beef fat was used as the lipid phase of the oleogel. Canola oil based oleogels produced softer meat batters.	[118]
Pastry	Hazelnut oil	5% sunflower wax or 5% beeswax	100% replacement of commercial bakery shortening.	[119]
	Canola oil	3% and 6% candelilla wax	30% replacement of cookie shortening with 3% candelilla wax, and 60% replacement with 6% candelilla wax.	[120]
	Sunflower oil	10% beeswax	Beeswax oleogel can replace 100% shortening in cakes. The cakes were high in unsaturated fat and low in saturated fats	[121]
	Canola oil	10% carnauba wax	25–50% shortening replacement in cakes.	[122]
	Palm stearin and soybean oil mixture	Ethylcellulose (1%, 2%, 3%, 4% or 6%) + 1% emulsifier	4% EC100 oleogel from palm stearin + soybean oil replaced bakery shortening totally in stable soft texture bread.	[123]
	Soybean oil	3% zein forming emulsion in glycerol with 0–0.045% β carotene fortification	Total replacement of bakery margarine in sponge cake, providing a similar texture between the oleogel-based cake and reference cake.	[124]
Dairy	High oleic sunflower oil	10% rice bran wax, candelilla wax, or carnauba wax	Rice bran wax had the best performance as a gelator in the production of ice cream. It brought about less fat destabilization and a greater meltdown rate.	[125]
	Sunflower oil	8% or 125% mixtures of phytosterols and γ-oryzanol	12% gelator produced better results and the ice cream obtained from the resultant oleogel showed similar or even better quality compared to the ice cream produced from regular milk cream.	[126]
	High oleic soybean oil	10% rice bran wax	Minimal degradation of vegetable oleogel cream cheese during storage was obtained as a result of thermal treatment.	[127]
Confectionery	Rice bran oil	1.5%, 2%, 2.5%, 3%, or 3.5% beeswax	17% of palm oil replacement with oleogel in hazelnut filling. Oil binding properties were also observed.	[128]
	Sunflower oil	10% or 25% mixtures of 1:1 γ-oryzanol and β-sitosterol	Oil migration was reduced by 50% when 2.5% or 14% of sunflower oil oleogels were layered into praline systems.	[129]
	Pomegranate seed oil	5% saturated monoglycerides, beeswax, and propolis wax	Oleogels replaced palm oil by 50% in chocolate spreads.	[130]
	Rapeseed oil	2% shellac wax	Oleogel provided an increase in oil binding capacity. 27% replacement of palm oil in chocolate pastes was also achieved.	[131]
Controlled release and delivery of bioactive compounds	Canola oil	2% of 12 hydroxistearic acid	Delivery of neutracetical or bioactive compounds via the release of β-carotene during digestion.	[132]

### 2.7. Physicochemical and Functional Properties of Oleogels

**Figure 7:**
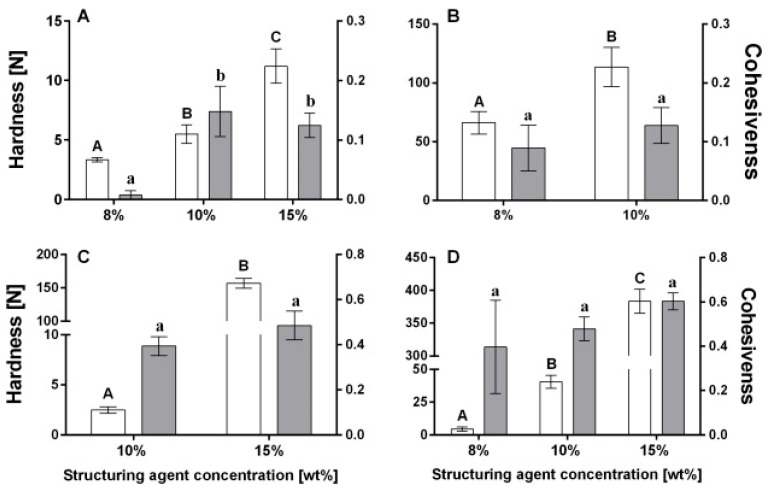
Hardness (white bars) and cohesiveness (gray bars) values obtained for oleogels based on (**A**) mono- and di-glycerides (E471), (**B**) β-sitosterol + γ-oryzanol, (**C**) ethylcellulose (EC 20 cP), and (**D**) ethylcellulose (EC 45 cP) with canola oil at various concentrations (wt%). The values followed by the same letter are not significantly different (*p* > 0.05). (Upper-case letters for the hardness and lower-case letters for the cohesiveness) [132].

▪ **Rheology and Texture Analysis**
  Because they affect texture, stability, and mouthfeel sense oleogels’ rheological characteristics are essential parameters to measure and study in order to design highly efficient candidates in food applications. The behavior of oleogels under various conditions and formulations has been clarified by studies using sophisticated rheological techniques, such as oscillatory shear rheology and texture profile analysis (TPA) [10,33,115]. Oleogels made with natural waxes were subjected to rheological profiling by Patel et al. in their 2015 study [81], which shed light on their stability and mechanical characteristics. The rheological behavior of oleogels is greatly influenced by variables such as the type and concentration of gelators, which makes it difficult to achieve uniform quality across food products. Oleogels have also been found to display thixotropic behavior, which improves their processing and handling capabilities [34]. Texture analysis is a really important area in evaluating oleogels, with instrumental methods used to quantify attributes such as firmness and spreadability, which are essential for consumer acceptance. Overall, there is potential for expanding the use of oleogels in the food industry through continued research into their rheological characteristics, texture analysis, and application. Oscillatory tests help confirm the gel-like structure of oleogels when the storage modulus (G’) exceeds the loss modulus (G”) [116]. The kind and concentration of oleogelators used are two of the many variables that affect the rheological performance of oleogels. LMWGs, for example, vary in their rheological and textural qualities according to their molecular structure and degree of hydration. Research findings have shown that, compared to higher viscosity grade polymer–MC 1500, a higher concentration of lower viscosity grade polymer, like MC 400, may result in better hydration and, as a result, improved emulsifying properties [73,91]. Thixotropic behavior, which describes oleogels’ capacity to regain elasticity and functionality over time following the removal of stress, has been noted. This characteristic is crucial for food applications because it improves the product’s usability and reduces the effects of mechanical handling. To measure this thixotropic behavior, particular tests are used, like the 3-ITT test, which assesses the oleogel’s structural deformation and regeneration under applied forces [34]. The rheological characteristics of oleogels are significantly influenced by their microstructure. Cryo-scanning electron microscopy (cryo-SEM) studies have demonstrated that the microstructure, which consists of densely packed droplet clusters scattered throughout an oil continuous phase, is largely unaltered following processing. This microstructure is greatly impacted by the shearing conditions and time; longer shearing times result in structural collapse and oil leakage. To preserve the intended rheological and textural characteristics of oleogels, shearing time and speed must be properly optimized [69,91,92]. An essential part of assessing the effectiveness and quality of oleogels used in food products is texture analysis. The mechanical and sensory properties of oleogels, which can replicate the actions of conventional fats like trans and hardstock fats in a variety of food matrices, are better understood thanks to this analysis. To evaluate important textural characteristics like firmness, stickiness, work of shear, and adhesiveness, instrumental texture analysis uses a variety of instruments. Rheology is applied in this context to shed light on the stability and flow characteristics of oleogels under various conditions [10,117]. For oleogels, where mechanical parameters are determined from force–time curves acquired during testing, Texture Profile Analysis (TPA) is especially pertinent. TPA curves that provide important details about the textural characteristics of oleogels can be produced using this technique [115]. Cohesion, adhesiveness, firmness, and spreadability are among the qualities that are measured in the analysis and are essential for product development and optimization [118]. A number of research studies have provided a good amount of rheological information on oleogels. For example, in a study [119] on the effect of the oleogelator type and its resultant structure on oil lipolysis and the bioaccessibility of the curcuminoids during in vitro digestion, Calligaris et al. rheologically characterized the oleogels produced. The oleogels all exhibited gel behavior, with the storage modulus (G’) being higher than the loss modulus (G”). In a 2018 [120] study, Wright and Marangoni investigated the phase behavior, microstructure, and rheology of vegetable oil-based ricinelaidic acid (REA) oleogels. Their results showed that the REA concentration influenced gelation kinetics and gel rheology, while temperature had a relatively less pronounced effect. Also, Wijarnprecha et al. [121] demonstrated that the rheology of water-in-oil emulsions may be tailored through oleogelation, particularly if the dispersed droplets are stabilized by interfacial crystalline shells that directly interact with the continuous phase gel network. In general, their study showed that even if oleogels may be less viscous than some gelled emulsions, they are less sensitive to shear.
▪ **Thermal Stability and Melting Behavior**
  Another crucial aspect of oleogel formulations for food products that must be carefully considered is their stability under varying temperature conditions. The thermal stability and melting behavior of oleogels are the main subjects of current research because these properties are essential to their functionality and acceptance in food formulations. For oleogels to remain intact and functional in a range of temperature conditions, their thermal stability is essential. According to studies, the kind and concentration of oleogelators have a big impact on the thermal characteristics of oleogels; some formulations are more stable than conventional fats [101,102]. For example, hydroxypropyl methylcellulose (HPMC)-based oleogels have shown exceptional resistance to thermal degradation, retaining their solid-like properties across a broad temperature range, which qualifies them for use in culinary applications where temperature swings are common. Additionally, stearic acid has been found to improve oleogels’ thermal stability, which results in increased firmness and oil-binding ability [77,124].
  Furthermore, studies using techniques like differential scanning calorimetry (DSC) have shed light on the melting profiles of oleogels and their distinct thermal properties [125,126]. Using a variety of experimental techniques, such as microscopy and rheological testing, numerous studies have assessed the thermal stability and melting behavior of oleogels. These studies have brought to light the different melting characteristics for varied oleogel formulations, as well as the crucial factors affecting their functionality, like yield stress and elastic moduli in thermal stress tests [77,127,128]. For oleogels to function well in food applications, their thermal stability is essential. The mechanical characteristics and structural behavior of oleogels under various thermal conditions have been investigated in recent advances in oleogel research. One important metric for assessing the stability and strength of oleogels is the oscillation yield stress, which is defined as the crossover point where the gel turns into a viscous sol [96,128]. According to amplitude sweep tests that measure elastic moduli under various conditions, a few studies [129,130] have shown that higher proportions of specific oleogelators can produce stronger gels and more stable networks. For oleogels to be used effectively in the food industry, they must be able to retain their integrity and structure under heat stress.
  The thermal stability of oleogels is a critical aspect of their performance in food applications. Oleogels are characterized by their ability to retain a gel network over a wide range of temperatures, which is particularly evident in HPMC-based oleogels that exhibit temperature-independent solid-like behavior, thus resisting thermal breakdown compared to traditional fats [77]. Techniques like the vial inversion technique, which evaluates the stability of oleogels under thermal conditions, can be used to confirm their formation [123]. The distinct melting behavior of oleogels’ structured oil systems is influenced by their composition and the interactions between different constituents. Oleogel melting profiles are frequently examined using Differential Scanning Calorimetry (DSC), which provides important information about the materials’ melting and thermal stability [125,126]. The solid fat content (SFC) of oleogels made of particular mixtures, such as monoglycerides, beeswax, and rice bran wax-based formulations, has been demonstrated to remain constant up to 45 °C in thermal stability tests. By testing oleogels over a temperature range of 20 to 160 °C, temperature sweep tests further clarify this stability by showing that they can tolerate heating without experiencing appreciable structural changes [131]. During thermal analysis, the melting profiles of different oleogel combinations, including beeswax–rice bran wax and beeswax–sunflower wax, have shown comparable behaviors. Higher-melting waxes (RBW and SW) melt at higher temperatures, after the low-melting BEW melts initially at lower temperatures. These findings demonstrate the behavior of monotectic phases, which include melting-induced changes from crystalline to liquid states. Moreover, peak melting temperatures for BEW do not change substantially with the presence of RBW or SW because they are constant across combinations [69]. These results imply that although the oleogels have unique melting properties, their overall melting performance is unaffected by the addition of particular waxes.
▪ **Oil Binding and Release Properties**
  Finally, another important feature that affects how well oleogels work in food applications is their ability to bind oil. In order to preserve the intended texture and sensory qualities of a variety of food products, studies have shown that oleogels can demonstrate high physical stability and a strong ability to retain oil [132] (Figure 7). As mentioned previously in this review, these binding characteristics have been characterized using methods like differential scanning calorimetry, rheological testing, and microscopy, which have shed light on how various gelators impact oil retention.
  The connection between oleogels’ mechanical strength and oil-binding ability is a crucial research topic. According to the existing literature, these two variables have a linear relationship, indicating that oleogels with tightly packed networks and greater mechanical strength can greatly reduce oil loss [12]. Because low oil binding capacity can result in undesired oil migration, which lowers product quality, this relationship is essential for preserving the textural and sensory aspects of food products [93,95]. The mechanisms via which oleogels regulate the release of oils during digestion are of significant interest. Research findings indicate that oleogels’ gel network may be able to control the rate at which lipids are released, which could result in a more gradual and long-lasting physiological reaction after consumption [96]. For instance, ethylcellulose has been shown to be an effective organogelator at structuring vegetable oils, which makes it a perfect choice for applications requiring regulated fat release [13].

### 2.8. Challenges and Limitations of Oleogels in the Food Industry

Despite the benefits, oleogels’ practical implementation is hindered by issues such as their sensory qualities and oxidative stability, which are crucial for consumer acceptance. Preserving the intended texture and functionality when using oleogels in place of conventional fats is still a major challenge that needs careful formulation and optimization. Further research is also required to fully understand how they interact with other food ingredients and how they behave under different processing and storage conditions [133].

▪ **Challenges in Oleogel Development**
  Achieving and preserving oleogel stability throughout processing and storage is one of the main stumbling blocks. An oleogel’s capacity to bind oil and withstand oxidative degradation is closely related to its stability. Oxidative stability is greatly influenced by variables like the type of oil used, storage temperature, and the presence of emulsifiers. Oleogels have demonstrated enhanced oxidative stability when made up of oils rich in monounsaturated fatty acids. This stability, however, is also dependent on the drying conditions used in the oleogel production [73,134]. Additionally, temperature changes, processing-related mechanical stress, and extended storage can cause instability in oleogels, which can damage their structure and cause oil syneresis [10,135]. Research has shown that slower cooling rates can improve gel phase stability by giving completely hydrated lamellar structures more time to form [93]. Furthermore, some oleogels may lose their structural integrity when exposed to high temperatures during cooking, while others can tolerate such conditions [117,135].
  The functional characteristics of oleogels, in particular their incorporation into various food matrices, present another major obstacle. For oleogels to be effective replacements, they must mimic the mouthfeel, texture, and stability of traditional fat-based systems [91]. To guarantee that the finished product satisfies sensory expectations, formulation and processing conditions must be well investigated. Formulation strategies can be complicated by the interactions between oleogels and other constituents, such as proteins and polysaccharides, which calls for a deeper comprehension of how these components function in different food applications [89,136]. Concerns have also been raised about potential detrimental alterations to technical and sensory attributes, including taste, color, and texture, that may occur when oleogel-based goods are processed and stored [137]. Gel stability and temperature resistance can be increased by using stabilizers like gellan gum; however, achieving the ideal ingredient balance to produce desired sensory qualities is still quite difficult [13,138,139]. Also, the complexity of oleogel structures may lead to variability in performance, which poses a challenge for consistent product quality [140].
▪ **Limitations of Oleogels in the Food Industry**
  The strict regulatory probing of food-grade gelators is one of the main challenges. Gelators must adhere to safety requirements and have their concentrations in food items limited by food laws [77,133]. These legal restrictions may restrict the use of specific oleogelators, making it more difficult to include them in food compositions [117]. Market adoption depends on adherence to food safety regulations, which may further limit the kinds of oleogelators that are permitted. The absence of commercial items is another constraint. There are currently no commercially accessible food products that use oleogels despite the encouraging outcomes of oleogel techniques and favorable sensory evaluations of goods containing oleogels. Due in large part to the difficulties in establishing economical and effective oleogelators appropriate for industrial use, this lack of market presence emphasizes the disconnection between research and real-world implementation [10]. There are difficulties with the technological aspects of producing oleogels, as well. Certain processing conditions are necessary for the formation of oleogels, and it is imperative to develop novel approaches that may effectively incorporate these gelators into food systems. Research on how different processing factors impact the quality of food products made using oleogel is still ongoing, but more work is needed to optimize these procedures for commercial scalability [77]. Another possible obstacle is consumer perceptions of food items that contain oleogels. Although natural polysaccharide gums have demonstrated potential as oleogelators because of their perceived consumer friendliness, oleogels’ widespread acceptability is still up in the air. Consumers’ desire to try new food additives may be impacted by unfavorable opinions about them, which calls for more research on their preferences and attitudes regarding the use of oleogel in food [69,117]. Lastly, cost issues are still an important factor in the development, acceptability, and widespread use of oleogels. The cost of sourcing and processing the necessary ingredients raises doubts about the current economic viability of oleogel production [70], and cost-effective solutions are required to make oleogels a viable option for widespread use in the food industry.

### 2.9. Future Perspectives and Research Directions

Due to continuous research and innovation, oleogels in the food industry are expected to see substantial developments in the future. Oleogels offer a possible substitute that can mimic the functional qualities of conventional fats while improving nutritional profiles, in light of the growing consumer demand for healthier food options and the regulatory concerns around trans fats. More research is required to completely comprehend the mechanisms driving oleogelation. Optimizing gelator combinations, improving processing methods, and investigating the potential health effects of oleogels in food products could be the main areas of research. Clinical studies investigating the metabolic impacts of oleogels in human diets, for example, may offer important new information about their potential uses. Furthermore, the creation of oleogels with customized melting points should improve their applicability in a range of food formulations, addressing challenges related to meltability and lipid release during digestion.

Oleogel production is becoming more efficient because of emerging technologies like the emulsion–template method created by Romoscanu and Mezzenga. In this multi-step procedure, an oil-in-water emulsion, stabilized by biopolymers, is created, and the water is then removed to create the appropriate gel structure [69,93]. Food manufacturers will have greater access to oleogels with customized qualities as a result of the potential for large-scale production, as researchers continue to improve these techniques. Food production is increasingly becoming more sustainable, and oleogels align with this trend. Oleogels, which can provide a plant-based substitute for conventional fats, can lessen the environmental effects of food production and the need for ingredients obtained from animals. The acceptability of oleogels in the market will depend on ongoing research into environmentally friendly production techniques and lifecycle assessment (LCA) studies.

Future research and development opportunities are presented by the rapidly changing field of oleogels in food technology. Improving knowledge of the sensory qualities and consumer acceptability of food products made with oleogels is one important avenue. Despite the fact that oleogels’ textural qualities have been described in a number of studies, there is still a significant lack of sensory assessments that can guide product development tactics. The difficulty of creating suitable sensory testing procedures while guaranteeing the safety of oleogel food products for ingestion may be the cause of this difficulty. Future research should concentrate on thorough sensory evaluation techniques that evaluate oleogel products’ taste, aroma, texture, and overall consumer perception in order to close these gaps. It is very important to address possible off-odors and flavors that have been identified in earlier studies. For instance, Bemer et al. (2016) [141] reported the detection of off-flavors and bitterness in oleogel cream cheese products, indicating an area that necessitates further investigation to enhance the overall sensory profile.

The industrial scalability of oleogel production is another crucial area that needs to be looked into. Evaluating the nutritional advantages, functional characteristics, and stability during the processing and storage of oleogels is necessary to determine their viability on an industrial scale. To ensure that oleogels can compete with conventional fats in the food industry, research should concentrate on improving production methods that can increase their economic viability. Additionally, improvements in oleogel formulation could be investigated, especially in terms of gelator types and concentration optimization. More desirable food products could result from research into innovative gelators that enhance the stability and sensory attributes of oleogels. The manufacture of products that live up to consumer expectations requires an understanding of how various ingredients interact and impact the textural and sensory qualities of oleogels. It is also critical to comprehend how customers behave when using products that contain oleogels. Research aiming at shedding more light on consumer attitudes, preferences, and propensity to use oleogel products may yield insightful information that informs product formulations and marketing tactics. Studies in this field could be used to pinpoint possible obstacles to adoption and guide tactics for successfully highlighting the advantages of oleogels in culinary applications.

## 3. Conclusions

Oleogels—structured oil-based systems—have garnered significant attention within the food industry due to their ability to replace solid fats and enhance the nutritional quality of diverse food products. Basically, the reason for this lies in the fact that the gel behavior arises when the storage modulus (G’) becomes higher than the loss modulus (G”). This is, in turn, a consequence of the structure-forming effect exerted by such agents at the nano and microscale. These systems, therefore, serve as versatile functional ingredients, capable of modifying texture, improving mouthfeel, and extending shelf life while reducing saturated fat content. Their utility is particularly notable in applications such as spreads, dressings, and baked goods, where they mimic the functional properties of conventional fats. The shift toward plant-based diets and healthier food alternatives has driven extensive research and development in oleogel technology. Consequently, a growing body of literature has emerged, focusing on the physicochemical properties, formulation strategies, and sensory performance of oleogels, with particular emphasis on their stability and applicability. Studies indicate that oleogels can be formulated using various gelling agents, including both natural and synthetic polymers, which influence their functionality in food applications. While oleogels show promise, the long-term health implications and consumer acceptance are still to be examined clearly. Certain gelling agents have raised questions about possible health effects, and regulatory challenges associated with novel food additives remain unresolved. Additionally, the sensory properties of oleogel-based products exhibit considerable variability, which may impact market adoption.

As a last take-home message, oleogels represent a promising approach to food reformulation, aligning with health trends and consumer demands, but further research is required to address existing limitations and optimize their integration into food systems. Ultimately, the successful adoption of oleogels in the food industry will depend on a balanced consideration of technological innovation, regulatory compliance, and consumer trust.

## Data Availability

No new data were created or analyzed in this study.
